# The Vaginal Microbiome is Associated with Endometrial Cancer Grade and Histology

**DOI:** 10.1158/2767-9764.CRC-22-0075

**Published:** 2022-06-16

**Authors:** Hesamedin Hakimjavadi, Sophia H. George, Michael Taub, Leah V. Dodds, Alex P. Sanchez-Covarrubias, Marilyn Huang, J. Matt Pearson, Brian M. Slomovitz, Erin N. Kobetz, Raad Gharaibeh, Ramlogan Sowamber, Andre Pinto, Srikar Chamala, Matthew P. Schlumbrecht

**Affiliations:** 1 Department of Pathology, Children's Hospital of Los Angeles, Los Angeles, California.; 2 Sylvester Comprehensive Cancer Center, Miami, Florida.; 3 Department of Obstetrics, Gynecology, and Reproductive Sciences, University of Miami Miller School of Medicine, Miami, Florida.; 4 Department of Obstetrics and Gynecology, Mount Sinai Medical Center, Miami, Florida.; 5 Department of Medicine, University of Miami Miller School of Medicine, Miami, Florida.; 6 Department of Medicine, University of Florida, Gainesville, Florida.; 7 Department of Pathology, University of Miami Miller School of Medicine, Miami, Florida.

## Abstract

The human microbiome has been strongly correlated with disease pathology and outcomes, yet remains relatively underexplored in patients with malignant endometrial disease. In this study, vaginal microbiome samples were prospectively collected at the time of hysterectomy from 61 racially and ethnically diverse patients from three disease conditions: (i) benign gynecologic disease (controls, *n* = 11), (ii) low-grade endometrial carcinoma (*n* = 30), and (iii) high-grade endometrial carcinoma (*n* = 20). Extracted DNA underwent shotgun metagenomics sequencing, and microbial α and β diversities were calculated. Hierarchical clustering was used to describe community state types (CST), which were then compared by microbial diversity and grade. Differential abundance was calculated, and machine learning utilized to assess the predictive value of bacterial abundance to distinguish grade and histology. Both α- and β-diversity were associated with patient tumor grade. Four vaginal CST were identified that associated with grade of disease. Different histologies also demonstrated variation in CST within tumor grades. Using supervised clustering algorithms, critical microbiome markers at the species level were used to build models that predicted benign versus carcinoma, high-grade carcinoma versus benign, and high-grade versus low-grade carcinoma with high accuracy. These results confirm that the vaginal microbiome segregates not just benign disease from endometrial cancer, but is predictive of histology and grade. Further characterization of these findings in large, prospective studies is needed to elucidate their potential clinical applications.

**Significance:**

The vaginal microbiome reliably segregates not just benign gynecologic condition from endometrial cancer, but also predicts cancer grade and histology. Patterns of microbial abundance and gene expression should be increasingly considered as a factor in the evolution of precision medicine approaches, especially as they relate to cancer screening, disease pathogenesis, and patient-centered outcomes.

## Introduction

Endometrial cancer is the most common gynecologic malignancy in the United States (1). The incidence of this disease has been increasing, and it is now listed as one of the leading causes of cancer death in women (1, 2). Aside from stratification by epidemiologic risk factors and genetic predisposition (3), there are no routine screening practices for endometrial cancer and women usually present when they develop symptoms. Unfortunately, knowledge of these symptoms is generally poor (4), potentially putting patients at risk for prolonged periods before oncologic evaluation.

While genomic classifications have been suggested to better distinguish between subtypes of endometrial malignancies (5), due to restrictions in cost and expediency required for treatment initiation, the histologic characterization of disease finds more clinical relevance in practice. Type I, or low-grade (LG) tumors, are driven by an overabundance of estrogen. These primarily glandular tumors with endometrioid histologies are commonly symptomatic early, diagnosed at earlier stages, and can in many cases be successfully treated with surgery alone. In contrast, type II, or high-grade (HG) tumors are characterized by aggressive presentations, often with metastatic disease at diagnosis as they may not be symptomatic in early stages (6). Comprised predominantly of serous, clear cell, carcinosarcoma, HG endometrioid, and undifferentiated histologies, type II malignancies have worse survival.

The human microbiome has been shown to influence cancer risk and outcomes by a number of mechanisms, including influencing inflammation, altering the genomic stability in host cells, and producing oncometabolites (7). Defining the microbiome by community state types (CST), which are groups of microbes of similar phyla and abundance, has been useful to describe differences across groups of women, but the association of CSTs with clinical and pathologic features in patients with endometrial cancer has not been described previously. Because endometrial cancer is a heterogeneous disease comprised of differing histologies and biologic drivers of malignant transformation, comparisons of microbial communities relative to specific histologies and grades may vary and suggest additional unexplored pathways for disease pathogenesis and propagation. Our primary objective was to conduct an exploratory analysis to characterize the preoperative vaginal microbiome in women undergoing surgery for endometrial cancer using metagenomic analyses. The secondary objective was to identify patterns which would reliably segregate not just benign from malignant disease, but also distinguish LG from HG tumors, as guided by CSTs. Such data may identify opportunities where further exploration of the microbiome in relation to disease pathogenesis or early detection is needed.

## Materials and Methods

### Ethical Approval and Consent

Approval for this study was provided by the Institutional Review Board at the University of Miami (Miami, FL; protocol no. 20170660). Informed consent was obtained from all participating patients, with forms provided in English, Spanish, and Haitian Creole. This cross-sectional study is reported in accordance with the Strengthening the Reporting of Genetic Association Studies reporting guideline (8). Patients were consented between February 2018 and October 2018 in a sequential manner without any preplanned stratification or matching. The initial protocol called for an oversampling of uterine serous carcinoma (planned accrual *n* = 10). Written informed consent was obtained from all patients, and the study was conducted in concordance with the Declaration of Helsinki.

### Population for Study and Patient-related Information

Three groups of patients were recruited for the study: (i) Women with benign gynecologic disease undergoing elective surgery for nonmalignant conditions, such as fibroids or endometriosis, and all with normal or inactive endometrium (controls); (ii) Women with LG endometrial carcinoma (EC), defined as endometrial intraepithelial neoplasia (EIN, preinvasive disease), grade 1 or grade 2 endometrioid adenocarcinoma on preoperative endometrial biopsy or uterine curettage; (iii) Women with HG endometrial carcinoma, defined as grade 3 endometrioid, serous, small-cell, clear-cell, undifferentiated, or dedifferentiated carcinoma, or uterine carcinosarcoma, on preoperative endometrial biopsy or uterine curettage.

Women were required to be ≥18 years of age, able to provide written consent, and able to read and understand English, Spanish, or Haitian Creole. All patients underwent surgery at one of the hospitals affiliated with the physician practice: University of Miami Hospital, Sylvester Comprehensive Cancer Center, or Jackson Memorial Hospital. Patients were excluded if they had an active gynecologic infection on physician assessment, had any contraindication to the introduction of a swab into the vagina (e.g., severe vaginal stenosis), administration of neoadjuvant chemotherapy, douching within 14 days of surgery, use of vaginal cream or lubricant within 14 days of surgery, use of antibiotics within 14 days of surgery, or sexual intercourse within 5 days of surgery.

Patient-specific information was collected from the electronic medical record, including: age at diagnosis, race, ethnicity, final histologic diagnosis [as determined by a gynecologic pathologist (A. Pinto)], tobacco use, body mass index (BMI), presence of lymphovascular space invasion (LVSI), and results of high-risk human papillomavirus (HPV) DNA testing from most recent Pap smear.

### Specimen Collection and Processing

On the day of surgery, following induction of anesthesia, and prior to both vaginal preparation with betadine/chlorhexidine and administration of prophylactic antibiotics, the vaginal swab (4N6FLOQSwab, Thermo Fisher Scientific, #4473979) was placed into the vagina by the attending physician, with care to ensure contact of the swab with the cervix, posterior fornix of the vagina, and vaginal sidewalls. The swab was immediately transferred to the bead tubes which were then snap frozen and kept at −80°C. Microbial DNA was extracted with the PureLink microbiome DNA purification kit (Thermo Fisher Scientific, Invitrogen, #A29790) following manufacturer's protocol. DNA was eluted in 50 μL of AE buffer and quantified using a NanoDrop 2000c Spectrophotometer (Thermo Fisher Scientific). Additional details regarding DNA library construction and sequencing can be found in Supplementary Data S1.

### Statistical Analyses

Statistical analyses were performed using custom scripts written in the statistical language R for Statistical Computing. To avoid bias, all patients were included in the analyses, even when missing specific data points, and all available data were included. Summary statistics were used to describe the entire cohort. Significant differences among patient clinical characteristics were determined using Kruskal–Wallis and Wilcoxon signed-rank test. All tests were two sided, with significance set at *P* < 0.05. Explanation of the power calculation can be found in Supplementary Data S1.

### Alpha and Beta Microbial Diversity

Alpha (α) and Beta (β) diversity are standard ecological measures of microbial diversity representing, respectively, the number of unique taxa per sample and similarity in composition between samples. We calculated the observed number of operational taxonomic units as the α-diversity measure for each sample within the tumor type groups after rarefaction. We also calculated the Shannon index as our main α-diversity metric, which was generally concordant with observed number of species. We then fitted a linear model for independent samples. The *t* test was used to determine statistical significance. For β-diversity, we rarefied the data prior to calculating the various distance measures. To test the association between the covariates and β-diversity measures, we used PERMANOVA, a distance-based analysis of variance method based on permutation. An omnibus test, which is a permutation test taking the minimum of the *P* values of individual β-diversity measures as the test statistic, was used to combine multiple sources of association evidence provided by different β-diversity measures and an overall association *P* value was reported. Ordination plots were generated using classic multidimensional scaling. Analyses of the effects of covariates are provided in Supplementary Data S2.

### Vaginal Community State Typing

Briefly, a matrix of sample dissimilarity was created based on the relative abundance of microbial species in each sample using Bray-Curtis distance method. CSTs were generated to classify the vaginal microbial communities, to explore community structure, and to reduce dimensionality based on previous reports (9, 10). Samples were clustered into four CSTs using the dissimilarity matrix as the input and Ward hierarchical clustering as the method, which minimized the total within-cluster variance. We used gap statistics to determine the optimum number of clusters in the dataset. Considering the sample size, we used *k* = 4 as the optimum number of clusters.

### Differential Abundance Analysis

We performed microbiome‐wide analysis to identify phylum, family, genus, and species that were differentially abundant between samples with different tumor grades and histology. Using phyloseq_to_deseq2 from phyloseq package (11), we transformed microbial relative abundance data into a DESeq dataset with dispersions estimated. We then identified differentially abundant taxa species using the Wald tests from R package DESeq2. We used samples’ species abundance without rarefying to account for variability in read depth between samples. Reported *P* values were adjusted for the FDR (*P*_adj_  <  0.05) using the Benjamini–Hochberg procedure.

### Gene Expression and Pathway Analysis

We used VIRGO (9) to identify and quantify community gene content, or gene richness, defined as the abundance of nonredundant genes. Nonredundant genes were also annotated with a rich set of functional descriptions. For gene set enrichment analysis (GSEA; ref. 12) we conducted enrichment analysis after constructing gene sets: overrepresentation and underrepresentation analyses across pathologies: benign, LG endometrial carcinoma, HG endometrial carcinoma, and tumor versus benign. We ranked genes based on their fold change (FC) between two sample groups using DEseq2 (13). Then using the fgsea R package, we performed GSEA with three gene sets including Kyoto Encyclopedia of Genes and Genomes (KEGG; ref. 14), Gene Ontology (15), and EggNOG, (v.5; ref. 16). Significantly enriched gene sets were filtered on the basis of a cutoff of *q* < 0.01.

### Machine Learning for Biomarker Discovery

Construction and evaluation of machine learning models on the basis of microbial species was performed using SIAMCAT (17). Read counts at the species level were converted to relative abundances. Species with an overall abundance lower than 0.01 were removed. To quantify associations between vaginal microbiome and tumor grade, we computed for each species the significance using Wilcoxon test and different effect sizes for the association (e.g., AUC or FC). The data used for feature selection were microbial relative abundance after the filtering of low abundant features. FDR was used to correct for multiple testing.

### Data and Materials Availability

All data associated with this study are available upon request and have been uploaded to Gene Expression Omnibus. SRA Submission ID: SUB9784683

## Results

### Demographics

The clinical and demographic characteristics of the studied cohort are displayed in Table 1.

**TABLE 1:** Clinical and demographic characteristics of the cohort^a^

	Benign (*n* = 11)	Low-grade EC (*n* = 30)	High-grade EC (*n* = 20)	
	Number (%)	Number (%)	Number (%)	*q*-value
Age at surgery	51.54 ± 10.77	60.00 ± 11.51	61.89 ± 10.46	0.024
Body mass index	30.99 ± 5.46	32.77 ± 7.09	37.30 ± 7.72	0.041
Tobacco use				0.652
Current	2 (3.4%)	4 (6.9%)	3 (5.2%)	
Former	2 (3.4%)	6 (10.3%)	1 (1.7%)	
Never	7 (12.1%)	18 (31%)	15 (25.9%)	
Human papillomavirus status				0.446
Negative	2 (3.4%)	15 (25.9%)	6 (10.3%)	
Positive	1 (1.7%)	1 (1.7%)	1 (1.7%)	
Unknown	8 (13.8%)	12 (20.7%)	12 (20.7%)	
Ethnicity				0.036
Hispanic	6 (9.8%)	17 (27.9%)	3 (4.9%)	
Non-Hispanic	5 (8.2%)	13 (21.3%)	17 (27.9%)	
Self-reported race				0.446
Asian	0	0	1 (1.7%)	
Black	2 (3.3%)	10 (16.7%)	10 (16.7%)	
White	9 (15%)	19 (31.7%)	9 (15%)	
Other	0	1 (1.7%)	0	

Abbreviation: EC, endometrial cancer.

^a^Parenthetical percentage are relative to entire study cohort. Because of missing data, percentages may not add up to 100%.

Patients with HG-EC were older than LG-EC and benign patients (*q* = 0.024). There was a significant difference in BMI between benign, HG-EC, and LG-EC patients (*q* = 0.041). More non-Hispanic patients were in the HG-EC cohort versus the LG-EC, in which there were more women of Hispanic ethnicity (*q* = 0.036). There were no differences in tobacco use, HPV status, or race across the three groups (all *P* > 0.05).

### Composition of the Vaginal Microbiome

Of the approximately 7.1 billion sequenced reads, 6.8 billion (95.1%) were identified as human sequences and removed from metagenomics analyses. Of the remaining 4.8% non-human sequence reads, 64% were taxonomically assigned known vaginal bacterial species (reads per sample shown in Supplementary Data S3). Taxonomic analysis revealed that our metagenomes contained 237 bacterial species with ≥ 0.01% relative abundance (of 273 previously described human bacterial species; ref. 17). The detected species included all major vaginal bacterial species (i.e., species from *Lactobacillus*, facultative, and strict anaerobic vaginal genus), as well as rare species (i.e., bacterial vaginosis-associated bacteria). The undetectable species included 37 rare species from *Mycoplasma, Staphylococcus, Taylorel, Chlamydophila, Chryseobacterium, Clostridium, Collinsella, Corynebacterium,* and *Dorea* genus.

**FIGURE 1:**
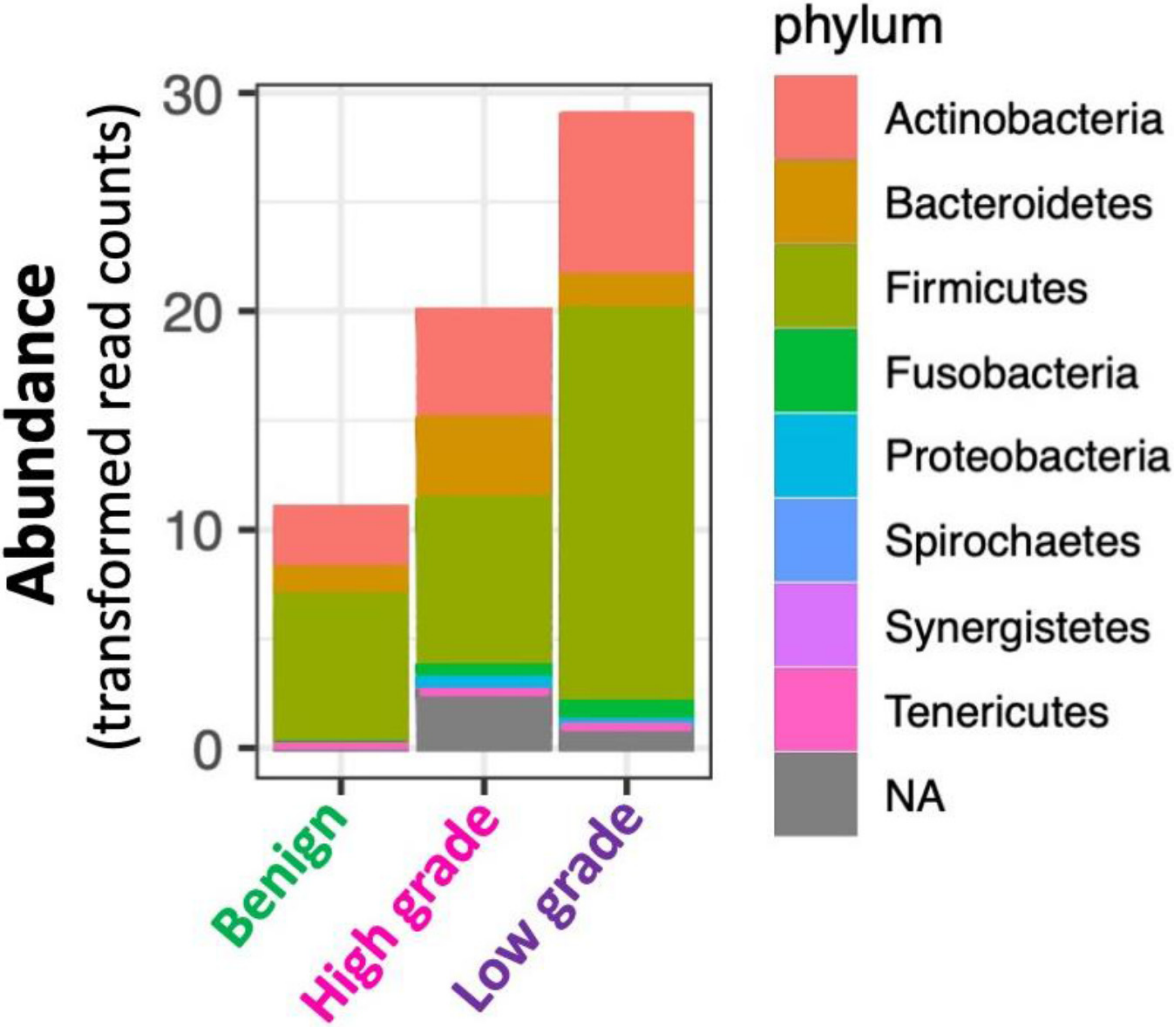
Differential phyla abundance across benign, LG endometrial cancer, and HG endometrial cancer (*P* = 0.093, across all three groups).

The most abundant phyla in all samples were *Firmicutes, Actinobacteria, and Bacteroidetes* (Fig 1). The most abundant species (based on total abundance over all samples) were *Gardnerella vaginalis*, *Lactobacillus iners*, *Streptococcus agalactiae*, and *Lactobacillus gasseri*. The most prevalent (proportion) species (present in all samples) were *Candidatus pelagibacter*, *Fusobacterium ulcerans*, *Gardnerella vaginalis*, and *Lactobacillus gasseri*. There was a significantly greater abundance of *Fusobacterium nucleatum* in HG relative to benign samples (log FC 4.3, *P* = 0.02); an increase in the abundance of *Fusobacterium nucleatum* was also seen in LG samples relative to benign, but was not significant (log FC 2.4, *P* = 0.066).

### Vaginal Microbiota Diversity

The vaginal microbiome was significantly different across α- (within samples) and β- (between samples) diversity in patients with the three disease conditions under investigation. Microbial α-diversity across all three groups was significant [ANOVA *P* values 0.024 (observed) and 0.032 (Shannon index)] (Supplementary Data S4). While the α-diversity of LG-EC patients was not significantly different from benign or HG patient samples, there was a significant increase in diversity from benign to HG disease (*P*_adj_ = 0.025). This trend suggests that HG disease coincides with a more diverse community of patient's vaginal microbiome.

We evaluated whether tumor types and other clinical factors—race, ethnicity, age, and BMI—were significant sources of β-diversity, which quantifies dissimilarities of microbial communities based on their composition. Samples grouped by clinical/demographic variables resulted in only one significant difference in microbial diversity. Meanwhile, one of the tumor related variables (tumor grade) resulted in significant *P* values (*P* = 0.042). Race (*P* = 0.273), ethnicity (*P* = 0.534), and BMI (*P* = 0.328) were not associated with β-diversity. This suggests that sample groups are more distinct in their microbial communities based on tumor-related factors than clinical/demographic factors.

#### CST Composition and Structure

**FIGURE 2:**
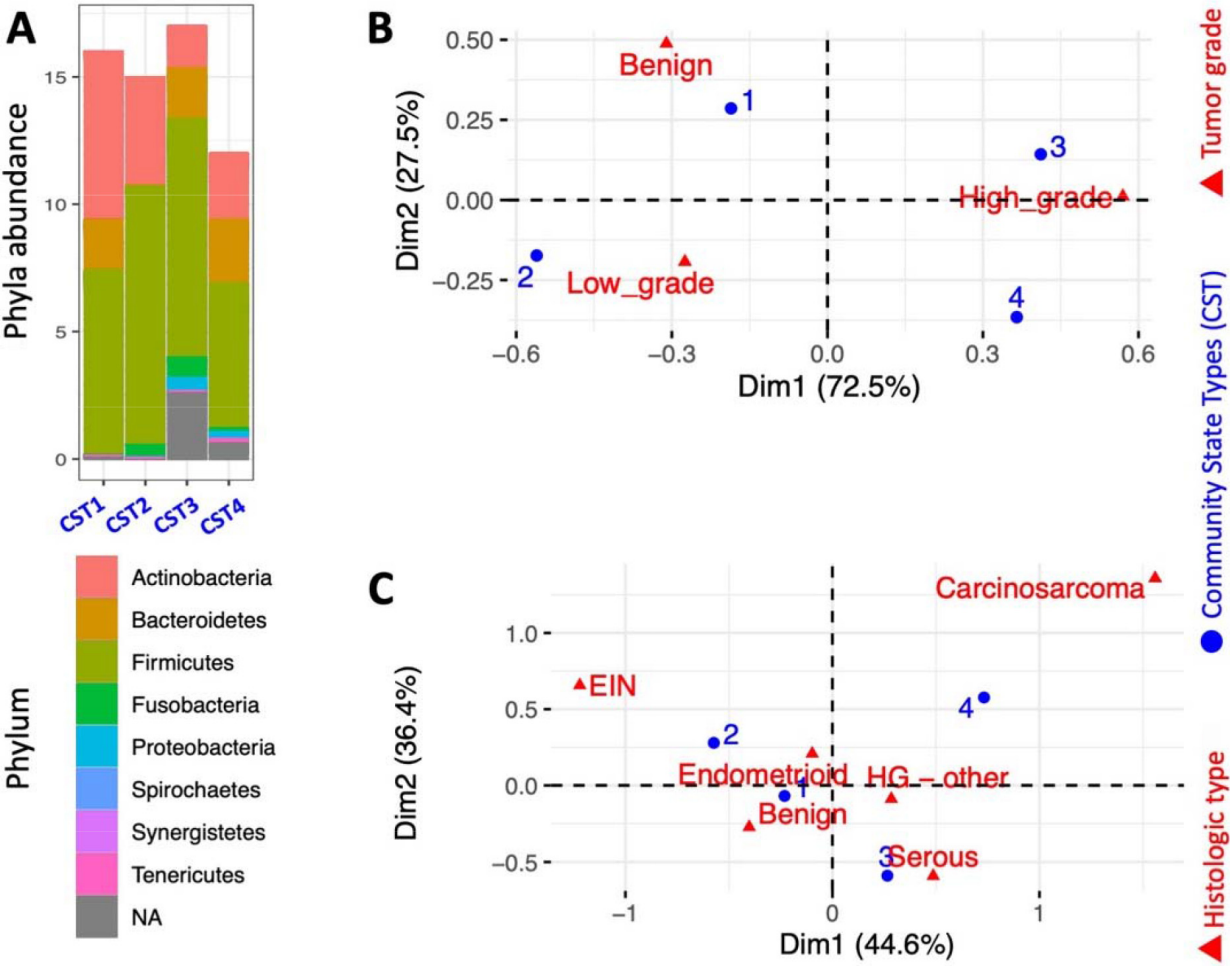
DA and CST structure. Differences were seen in the microbial phyla abundance by CST (**A**). CSTs were also significantly associated with both tumor grade (**B**, *P* = 0.036) and tumor histology (**C**, *P* = 0.017).

Four major CSTs were identified with significant differences in microbiome composition, diversity, and structure. Each of the four identified CSTs was comprised of communities disproportionately composed by different phyla (Fig 2A). *Bacteroidetes* was absent in CST2, and *Fusobacteria* absent in CST1. *Acinetobacteria* and *Firmicules* were variably present across all four CSTs. The most diverse and taxonomically rich cluster was CST4; the least was CST2.

There was statistically significant clustering into CSTs by both grade and histology (Fig 2B and C). Benign disease predominantly clustered in CST1, while LG clustered in CST2, and HG into both CST3 and CST4 (*P* = 0.036). There was also variation in CST clustering by histology (*P* = 0.017). Clinical characteristics and CSTs were evaluated against microbial diversity; only grade and histology had significant associations (benign vs. HG, *P*_adj_ = 0.019; benign vs. carcinosarcoma, *P*_adj_ = 0.037; benign vs. EIN, *P*_adj_ = 0.037; Table 2).

**TABLE 2:** Microbial diversities of samples based on clinical variables

Variable	Comparison	*P*
Age	<50 years (ref)	
	50–69	0.13
	> = 70	0.39
Race	White (ref)	
	Black	0.36
	Asian	0.61
	Other	0.61
Ethnicity	Non-Hispanic (ref)	
	Hispanic	0.058
BMI	<25 (ref)	
	25−<30	0.69
	30−<35	0.94
	35−<40	0.91
	> = 40	0.96
Tumor grade	Benign (ref)	
	Low-grade	0.467
	High-grade	0.019
Histology	Benign histology (ref)	
	EIN	0.037
	Endometrioid	0.229
	Serous	0.068
	Carcinosarcoma	0.037
	Other high-grade	0.116
LVSI	Absent (ref)	
	Present	0.45

### Differential Abundance Analysis

**FIGURE 3:**
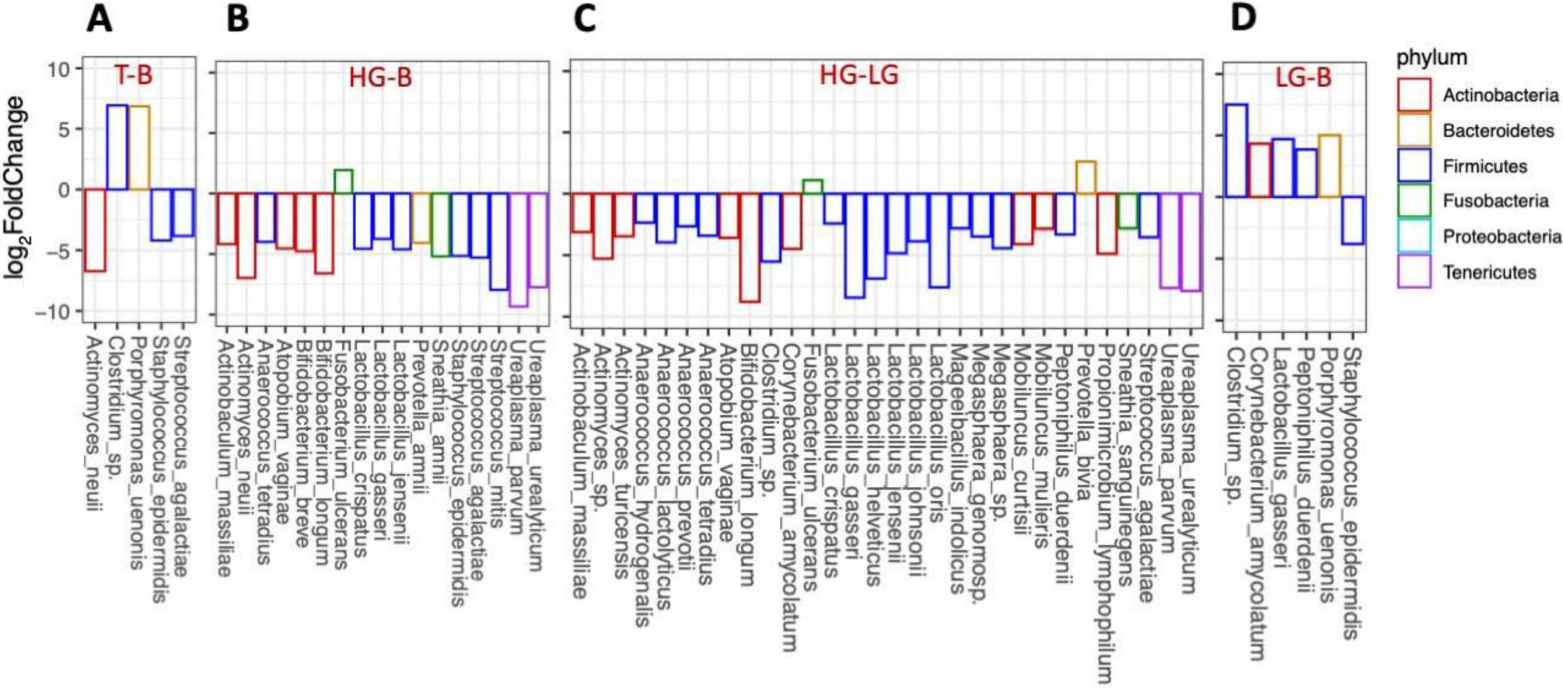
DA by tumor grade. Positive FC indicates enrichment of species, whereas negative FC indicates paucity of species. In tumors (including both HG and LG), Clostridium sp. and Porphyromonas uenonis are more abundant compared with benign (**A**). Fusobacterium ulcerans is the only species significantly more abundant in HG compared with benign (**B**). Similarly, Fusobacterium ulcerans and Prevotella bivia are the only two species significantly more abundant in HG compared with LG endometrial cancer (**C**). LG endometrial cancer metagenomes versus benign have distinct abundance of Clostridium sp. Corynebacterium amycolatum, Lactobacillus gasseri and Peptoniphilus duerdeni, including Porphyromoas uenonis (**D**). Only taxa with significant changes in abundance are shown (*P*_adj_ < 0.05, Wald test).

Differential abundance (DA) analyses were conducted to determine the vaginal microbial species enriched or depleted consistently in EC communities. The comparison of relative abundance between benign versus tumor (LG + HG) revealed that profiles obtained from tumor have only five species with statistically significant DA relative to benign samples (*P*_adj_ < 0.05, Wald test; Fig 3; Supplementary Data S5). Dividing tumor samples into LG and HG profiles identified 30 DA species between HG and LG as well as 17 DA species between HG and benign samples. Noticeably, the abundance of 46 species is significantly lower in the HG sample compared with other sample groups. However, two species (*Fusobacterium ulcerans* and *Prevotella bivia*) were found with higher abundance in HG samples. Between LG and benign groups, there were five species with significantly greater abundance in the tumors; only *Staphylococcus epidermidis* demonstrated lower abundance.

### Gene Expression and Pathway Analyses

The metagenomic approach used allows us to investigate gene abundance and thus pathway analyses of the microbiota observed across endometrial pathologies and endometrial cancer histotypes. The HG communities were typically categorized as low gene count as 73.8% of them had less than 1,000 genes. Benign communities commonly displayed high gene count as 65% of them had more than 1,000 genes. Hierarchical clustering of the profiles was performed using ward linkage based on their Euclidean distance, the result of correspondence analysis conducted for gene richness and diagnosis. We found a strong dependence between the three gene-based clusters and the three tumor grades (*P* = 0.025, *χ*^2^ test). The gene-based clusters, however, were independent of other clinical variables including race, ethnicity, BMI groups, age, disease stage, and menopause status (*P*: 0.64, 0.37, 0.37, 0.08, 0.22, and 0.22, respectively; Supplementary Data S6 and S7).

Using VIRGO, each nonredundant gene was taxonomically and functionally annotated. We next identified significant associations (FDR *P* < 0.05) between microbial abundance, gene family, and pathway abundance first across tumor and benign, and then more specifically across benign, LG-EC and HG-EC. KEGG pathway analysis of tumor versus benign had the highest number of *P* < 0.05 statically significant associations. Purine metabolism and ATP-binding cassette (ABC) transporter pathways were upregulated in tumors whereas genes associated with viral myocarditis, aminoacyl-tRNA biosynthesis, and glutathione metabolism were downregulated in the endometrial tumor microbiota (Supplementary Data S8A). Additional analyses of the metagenome of HG endometrial cancers alone compared with benign revealed the only pathway significantly upregulated is biosynthesis of siderophore group nonribosomal peptides. Conversely, pathways downregulated included the pyrimidine metabolism, purine metabolism, homologous recombination, and ABC transporters (Supplementary Data S8B). The downregulation of gene sets in homologous recombination, mismatch repair and ABC transporters was unique to HG-EC microbiota.

### Biomarker Discovery

**FIGURE 4:**
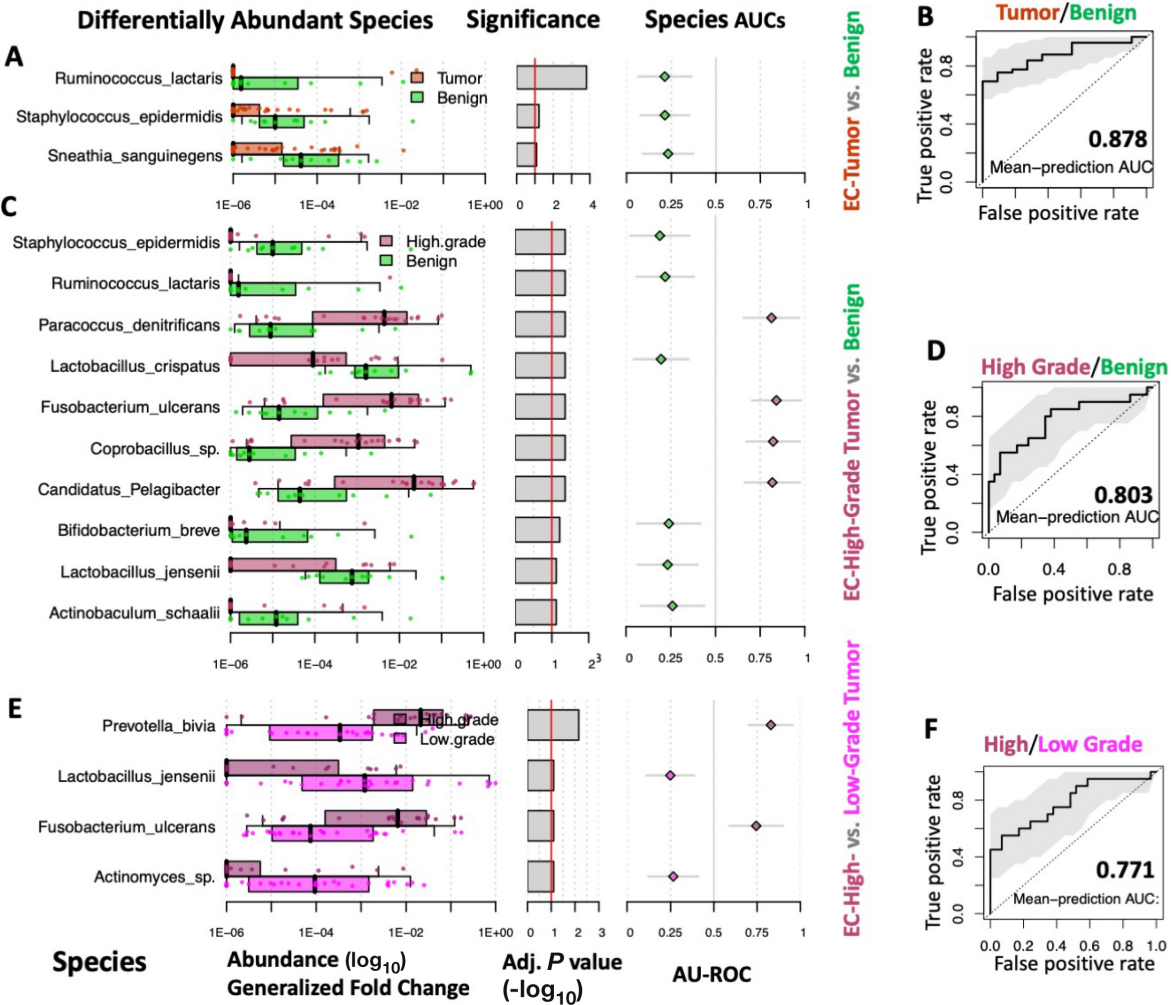
Biomarker discovery by grade. Validation was performed on random forest classifier models, which identified an optimal microbiome signature for each cohort (**A**, **C**, **E**). These signatures were used to construct receiver operating curves which discriminate benign versus tumor (**B**), HG versus benign (**D**), and HG versus LG (**F**).

To examine the diagnostic value of the vaginal microbiome, we constructed random forest (RF) models that could specifically classify samples according to patients’ tumor types including (i) benign versus tumor samples, (ii) HG tumors versus benign, and (iii) HG tumors versus LG tumors (Fig 4). To detect useful species markers of tumor, we conducted a fivefold cross-validation on a random forest model between case and control samples in the discovery phase. For each model, a different set of species was identified as an optimum microbiome signature, consisting of a various number of features and performance of the constructed models based on the area under the ROC curve (Fig 4A–E). The tumor versus benign model selected three important species. The discriminant model based on the abundance of these species effectively distinguishes tumor from benign disease (mean prediction AUC = 0.878; Fig 4B). Two other RF models generated from additional species abundance distinguished HG from benign, and LG from HG with AUC of 0.80 and 0.77, respectively (Fig 4D–F).

We examined the performance of models trained by samples labeled according to histologic subtype (e.g., serous, endometrioid;Supplementary Data S9). The highest prediction performance obtained from the model that trained to distinguish benign samples from samples labeled as serous endometrial carcinoma (mean AUC = 0.826) followed by two models that distinguish benign from endometrioid samples (mean AUC = 0.795) and serous from endometrioid (mean AUC = 0.776). Each of these three histologic classifier models is based 50, 60, and 65 biomarker species, respectively.

## Discussion

Among patients with endometrial carcinoma, the vaginal microbiome demonstrates significant variation by tumor pathologic characteristics. This exploratory investigation establishes that not only do prominent species vary by grade, but so too do microbial abundance and CST. These findings represent a novel perspective on the microbial content of the vagina and how the confluence with the uterus may provide opportunities for further exploration into its role as an indicator of endometrial carcinoma or further understanding of disease development and propagation.

There have been few studies about the vaginal microbiome in patients with endometrial carcinoma. In 2016, Walther-Antonio and colleagues (18) assessed the microbiome (16S) of the entire gynecologic tract of 17 patients with endometrial carcinoma and 10 with benign uterine conditions. The authors reported that the microbiome across different gynecologic sites was significantly correlated, suggesting that vaginal sampling is an accurate surrogate of the microbiome within the uterus. Subsequent investigations have also confirmed that the vaginal microbiome mirrors that of the upper genital tract among women with cancer (19). In addition, it was reported that the pattern of presence or absence of *Porphyromonas* and *Atopobium* species was predictive of endometrial carcinoma (AUC 0.90; ref. 18). Within our cohort, neither of these phyla demonstrated significant abundance. In contrast to the Walther-Antonio study, however, we assessed specific tumor grade relative to benign conditions. Additionally in their study, all patients with endometrial carcinoma were White; 37% of our population was Black. Microbial diversity has been shown to be greater in Black versus White women (20), and CST in premenopausal women defined by Lactobacillus varies across all races (Black vs. White vs. Asian; ref. 21), so the differences in the populations between our two studies may account for the discrepancy.

A follow-up study by Walsh and colleagues which included 56 patients with endometrioid histology and 10 with nonendometrioid, HG, histology, also reported that *Porphyromonas somerae* was a predictive biomarker in endometrial carcinoma, and that additional sensitivity to disease detection was added by including patient-specific factors such as BMI, vaginal pH, and menopausal status (19). While we did not assess vaginal pH in the current study, we found no association between age or BMI and microbial diversity (Table 2). Our methodology, however, differed in that our data were segregated categorically to represent clinically meaningful groups (i.e., BMI following World Health Organization categorization; age of 50 serving as a surrogate for menopause). This variation in analysis may account for our findings, but could also be reflective of differences in the population of study relative to our own, as 97% of the Walsh cohort was White and only 10 patients had HG cancers. As microbial diversity in the current study was associated with tumor factors only (grade and histology) and not with categorical clinical factors, it suggests that patient-specific factors may not necessarily need to be included in a predictive model for screening.

While the differential phyla abundance between benign and tumor provides some insight into the local vaginal environment, differences in species abundance may also be meaningful in terms of tumor pathogenesis. *Prevotella bivia*, with greater than a 6-fold abundance in HG versus LG, is associated with pelvic inflammatory disease and bacterial vaginosis. *P. bivia* has been shown to upregulate proinflammatory (LAMP3, STAT1, and TAP1) genes in cervical cancer (22). Furthermore, Lactobacillus spp, which were underrepresented in HG versus benign and HG versus LG, are known to inhibit *P. bivia* (23). *Bifidobacterium longum* was the most greatly suppressed species in terms of abundance in HG versus LG disease. *B. longum* has been shown to have low relative abundance in patients with the most aggressive forms of gastric cancer, suggesting it may be protective (24). It has also been shown to improve immune-mediated tumor control (25). *Fusobacterium ulcerans* also demonstrated higher abundance in HG. This species has an association with cellular ulceration by secretion of high levels of butyrate (26); very little data exist about its role in cancer pathogenesis. *Fusobacterium nucleatum,* though not one of the most abundant species contributing to the predictive models, but with a greater than 4-fold presence in HG versus benign, has been found to promote tumor growth (27), associate with high microsatellite instability (28), and induce chemotherapy resistance (29). Patients with cervical cancer who have high levels of intratumoral *F. nucleatum* have worse progression-free and overall survival (30). In colorectal cancer, the bacterium secretes the adhesin Fap2, which binds to galactose *N-*acetyl-D-galactosamine (Gal-GalNAc), facilitating the enrichment of tumor cells (29). Gal-GalNAc levels have been shown to be higher in uterine adenocarcinomas relative to benign endometrium (31), and overexpression of the transferases that facilitate Gal-GalNAc glycosylation are strongly associated with histologic grade of tumor and myometrial invasion (32). In colorectal cancer cells *in vitro*, a high abundance of intratumoral *F. nucleatum* also activates autophagy, thus inducing resistance to platinum-based chemotherapy (29). The role of all these bacteria in the pathogenesis and treatment of endometrial carcinoma, and specifically high-grade histologies, requires further investigation.

The mechanisms by which the microbiome influences endometrial carcinoma pathogenesis have yet to be determined but are likely multifactorial in the context of tumor stromal function and alterations in cancer cell signaling pathways. Lu and colleagues recently reported that the presence of specific bacteria in the endometrium are associated with variable levels of the proinflammatory cytokines IL6, IL8, and IL17 (33). These molecules are known to modify the local microenvironment, and have been implicated in gynecologic cancer development through increased angiogenesis, cellular proliferation, and modification of local immune response (34–36). In patients with colorectal cancer, the presence of *F. nucleatum*, may activate the Wnt/β-catenin signaling pathway (37). In the endometrium, this pathway is important for normal physiologic cellular proliferation during the menstrual cycle, but oncogenic activation is also associated with endometrial carcinoma development (38, 39). Consideration should also be given to environmental mediators of microbial content, as practices such as douching have also been shown to favorably modify the gynecologic tract for pathogens (40).

There are several limitations to our study. Our population was from a single institution, so the results may not be applicable in other study environments. Nonetheless, the population was racially and ethnically diverse, which may increase generalizability. Though our sample population was small, we were still able to identify statistically significant associations between CSTs and histology, with >90% power (Supplementary Data S1). Additionally, these relationships were maintained across our analyses, including composition and DA. We designed the study to specifically include more patients with serous carcinoma, and this oversampling approach allowed for greater representation of understudied, high-risk endometrial histologies, relative to other reports (18, 19). Moreover these analyses used a metagenomics approach instead of 16S rRNA sequencing in the assessment of endometrial carcinoma–associated microbiomes. This allowed for a more robust evaluation of relative microbial abundance and diversity. While others have advocated for the use of one or two species to discriminate between benign and malignant (18, 19), this study included multiple bacterial species to define clusters of organisms that collectively predicted not just malignancy, but subsets of disease. Such an approach may increase the accuracy of these models. Further increases in model accuracy may be achieved with inclusion of tumor-specific factors that can affect bacterial milieu, such as tumor size/volume, degree of myometrial invasion, and amount of necrosis, which were not utilized as covariates in the current investigation.

## Conclusions

In this exploratory analysis, the vaginal microbiome reasonably segregated not only endometrial carcinoma from benign disease, but also had strong potential predictive value by grade and histology. Further study in larger populations is needed for validation of our findings, with continued attention to diverse populations to capture variations that may arise from differences associated with clinically relevant demographic factors (race, ethnicity, immigrant status, etc.). The role of the microbiome as a biomarker of disease requires additional exploration, especially because endometrial carcinoma is a disease for which no tool exists for screening or early detection. It will also be important to further characterize the relationships between the microbiome and tumor microenvironment, be they symbiotic or simply associative, and how these may contribute to disease etiology, tumor propagation, and potential novel therapeutic approaches.

## Supplementary Material

Supplement 1 Additional statistical methods

Supplement 2 Analysis of Confounders

Supplement 3 Individual Specimen Reads

Supplement 4 Microbial alpha diversities

Supplement 5 Microbial abundance by histotype

Supplement 6 Heatmap of protein functions

Supplement 7 Heatmap of abundance values

Supplement 8 Functional pathway analysis

Supplement 9 Biomarker discovery by histology
