# Supplementary material for: The Vaginal Microbiome is Associated with Endometrial Cancer Grade and Histology
Source: Cancer Res Commun. 2022 Jun 16;2(6):447–55. doi: 10.1158/2767-9764.CRC-22-0075 (PMC9345414; doi:10.1158/2767-9764.CRC-22-0075)
Supplement: Supplement 3 — Individual Specimen Reads [file crc-22-0075-s03.docx]

| Sample | Total read counts | Non-human read counts recovered | Non-human reads ratio | Read counts recovery levels |
| --- | --- | --- | --- | --- |
| B1 | 117,225,578 | 51,087,211 | 43.58% | High |
| B10 | 113,715,875 | 763,021 | 0.67% | Low |
| B11 | 119,903,716 | 2,027,020 | 1.69% | Medium |
| B12 | 121,244,886 | 17,335,769 | 14.30% | High |
| B2 | 118,174,292 | 960,695 | 0.81% | Low |
| B3 | 122,308,400 | 922,304 | 0.75% | Low |
| B5 | 117,163,218 | 8,373,898 | 7.15% | Medium |
| B6 | 115,445,964 | 6,560,886 | 5.68% | Medium |
| B7 | 122,056,396 | 957,284 | 0.78% | Low |
| B8 | 119,670,110 | 5,389,244 | 4.50% | Medium |
| B9 | 115,569,598 | 44,173,611 | 38.22% | High |
| HG1 | 117,982,636 | 797,127 | 0.68% | Low |
| HG10 | 122,526,058 | 895,529 | 0.73% | Low |
| HG11 | 118,539,310 | 887,929 | 0.75% | Low |
| HG12 | 126,095,316 | 13,609,923 | 10.79% | High |
| HG13 | 123,999,612 | 1,047,033 | 0.84% | Low |
| HG14 | 121,828,702 | 1,007,314 | 0.83% | Low |
| HG15 | 119,171,082 | 5,710,525 | 4.79% | Medium |
| HG16 | 112,800,494 | 59,678,007 | 52.91% | High |
| HG17 | 121,746,994 | 3,666,129 | 3.01% | Medium |
| HG18 | 119,204,954 | 740,087 | 0.62% | Low |
| HG19 | 119,153,176 | 724,762 | 0.61% | Low |
| HG2 | 117,568,254 | 3,421,745 | 2.91% | Medium |
| HG20 | 121,852,888 | 803,323 | 0.66% | Low |
| HG21 | 116,681,674 | 651,422 | 0.56% | Low |
| HG3 | 120,177,322 | 819,797 | 0.68% | Low |
| HG4 | 120,110,320 | 1,119,478 | 0.93% | Low |
| HG5 | 122,498,220 | 913,254 | 0.75% | Low |
| HG7 | 118,005,566 | 778,810 | 0.66% | Low |
| HG8 | 122,303,372 | 844,752 | 0.69% | Low |
| HG9 | 118,043,008 | 780,256 | 0.66% | Low |
| LG1 | 119,744,876 | 1,500,215 | 1.25% | Medium |
| LG10 | 119,592,652 | 861,072 | 0.72% | Low |
| LG11 | 121,589,928 | 763,508 | 0.63% | Low |
| LG12 | 116,545,860 | 3,919,445 | 3.36% | Medium |
| LG13 | 121,390,048 | 2,913,206 | 2.40% | Medium |
| LG14 | 121,166,050 | 3,462,731 | 2.86% | Medium |
| LG15 | 121,152,664 | 705,079 | 0.58% | Low |
| LG16 | 122,488,516 | 9,203,419 | 7.51% | Medium |
| LG17 | 119,053,156 | 1,554,269 | 1.31% | Medium |
| LG18 | 119,294,218 | 986,602 | 0.83% | Low |
| LG19 | 116,528,254 | 21,827,890 | 18.73% | High |
| LG2 | 115,033,174 | 845,553 | 0.74% | Low |
| LG21 | 120,133,742 | 1,538,275 | 1.28% | Medium |
| LG22 | 119,108,594 | 10,474,079 | 8.79% | Medium |
| LG23 | 119,786,446 | 858,174 | 0.72% | Low |
| LG24 | 115,705,276 | 2,753,446 | 2.38% | Medium |
| LG26 | 122,933,868 | 875,173 | 0.71% | Low |
| LG27 | 118,785,162 | 10,767,642 | 9.07% | Medium |
| LG29 | 115,900,338 | 5,845,843 | 5.04% | Medium |
| LG3 | 119,884,970 | 1,071,121 | 0.89% | Low |
| LG30 | 119,001,506 | 930,740 | 0.78% | Low |
| LG31 | 118,865,246 | 926,118 | 0.78% | Low |
| LG32 | 119,097,594 | 4,268,742 | 3.58% | Medium |
| LG33 | 121,552,554 | 10,885,799 | 8.96% | Medium |
| LG5 | 119,828,210 | 3,438,813 | 2.87% | Medium |
| LG6 | 117,987,812 | 33,373,560 | 28.29% | High |
| LG7 | 119,830,216 | 1,407,805 | 1.18% | Medium |
| LG8 | 117,533,206 | 904,485 | 0.77% | Low |
| LG9 | 117,554,892 | 20,787,150 | 17.68% | High |
