# Supplementary material for: The Vaginal Microbiome is Associated with Endometrial Cancer Grade and Histology
Source: Cancer Res Commun. 2022 Jun 16;2(6):447–55. doi: 10.1158/2767-9764.CRC-22-0075 (PMC9345414; doi:10.1158/2767-9764.CRC-22-0075)
Supplement: Supplement 4 — Microbial alpha diversities [file crc-22-0075-s04.docx]

**Supplement 4:** Microbial α-diversities across samples of benign control, low-grade endometrial cancer, and high-grade endometrial cancer.

|  | ANOVA P.Val | HG vs B p.adj | LG vs B p.adj | LG vs HG p.adj |
| --- | --- | --- | --- | --- |
| observed | 0.024* | 0.025 | 0.481 | 0.112 |
| Diversity Shannon | 0.032* | 0.054 | 0.810 | 0.068 |
