# Supplementary material for: The Vaginal Microbiome is Associated with Endometrial Cancer Grade and Histology
Source: Cancer Res Commun. 2022 Jun 16;2(6):447–55. doi: 10.1158/2767-9764.CRC-22-0075 (PMC9345414; doi:10.1158/2767-9764.CRC-22-0075)
Supplement: Supplement 5 — Microbial abundance by histotype [file crc-22-0075-s05.docx]

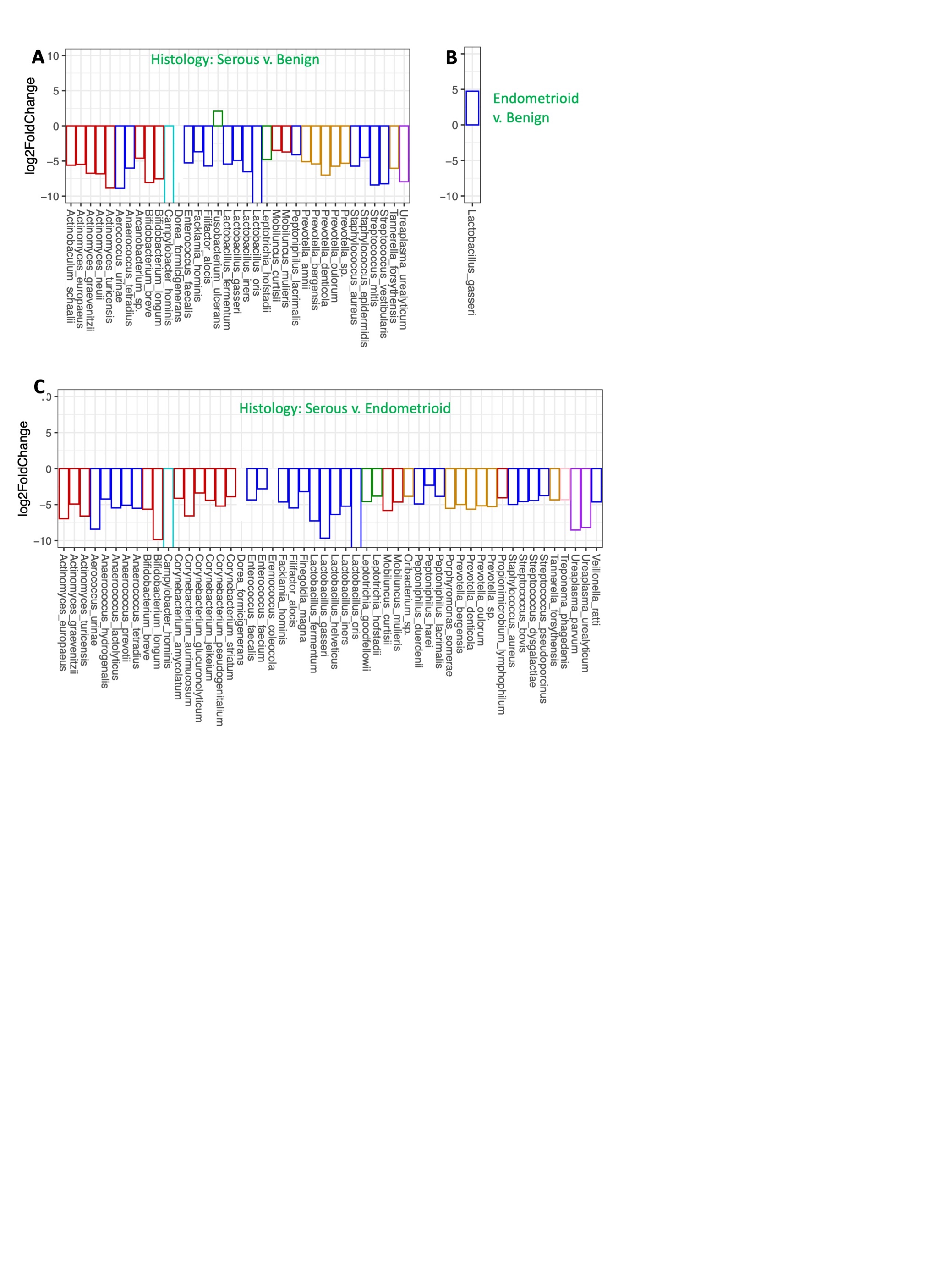


**Supplement 5.** Microbial abundance by histotype. Only taxa with significant changes in abundance are shown in the figure (adjusted p.value<0.05, Wald test). **A**. Serous versus benign. **B**. Endometrioid vs benign. **C**. Serous versus endometrioid.
