# Supplementary material for: The Vaginal Microbiome is Associated with Endometrial Cancer Grade and Histology
Source: Cancer Res Commun. 2022 Jun 16;2(6):447–55. doi: 10.1158/2767-9764.CRC-22-0075 (PMC9345414; doi:10.1158/2767-9764.CRC-22-0075)
Supplement: Supplement 6 — Heatmap of protein functions [file crc-22-0075-s06.docx]

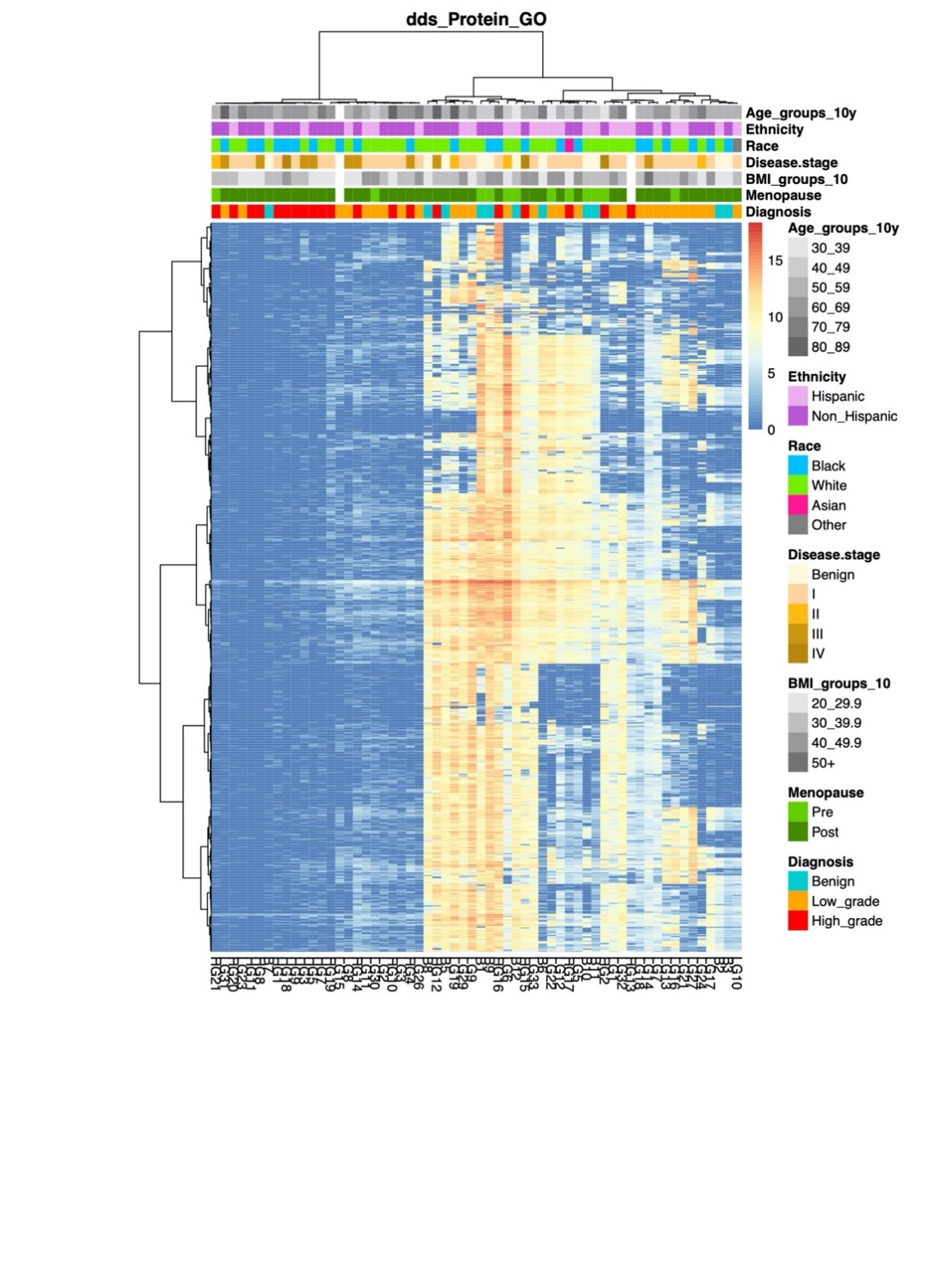


**Supplement 6.** Heatmap of protein functions enrichment score for 61 vaginal metagenomes (red: high enrichment, blue: low enrichment). Functional categories were defined using protein Gene Ontology (proteinGO). Hierarchical clustering of the profiles was performed using ward linkage based on their Euclidean distance.
