# Supplementary material for: The Vaginal Microbiome is Associated with Endometrial Cancer Grade and Histology
Source: Cancer Res Commun. 2022 Jun 16;2(6):447–55. doi: 10.1158/2767-9764.CRC-22-0075 (PMC9345414; doi:10.1158/2767-9764.CRC-22-0075)
Supplement: Supplement 8 — Functional pathway analysis [file crc-22-0075-s08.docx]

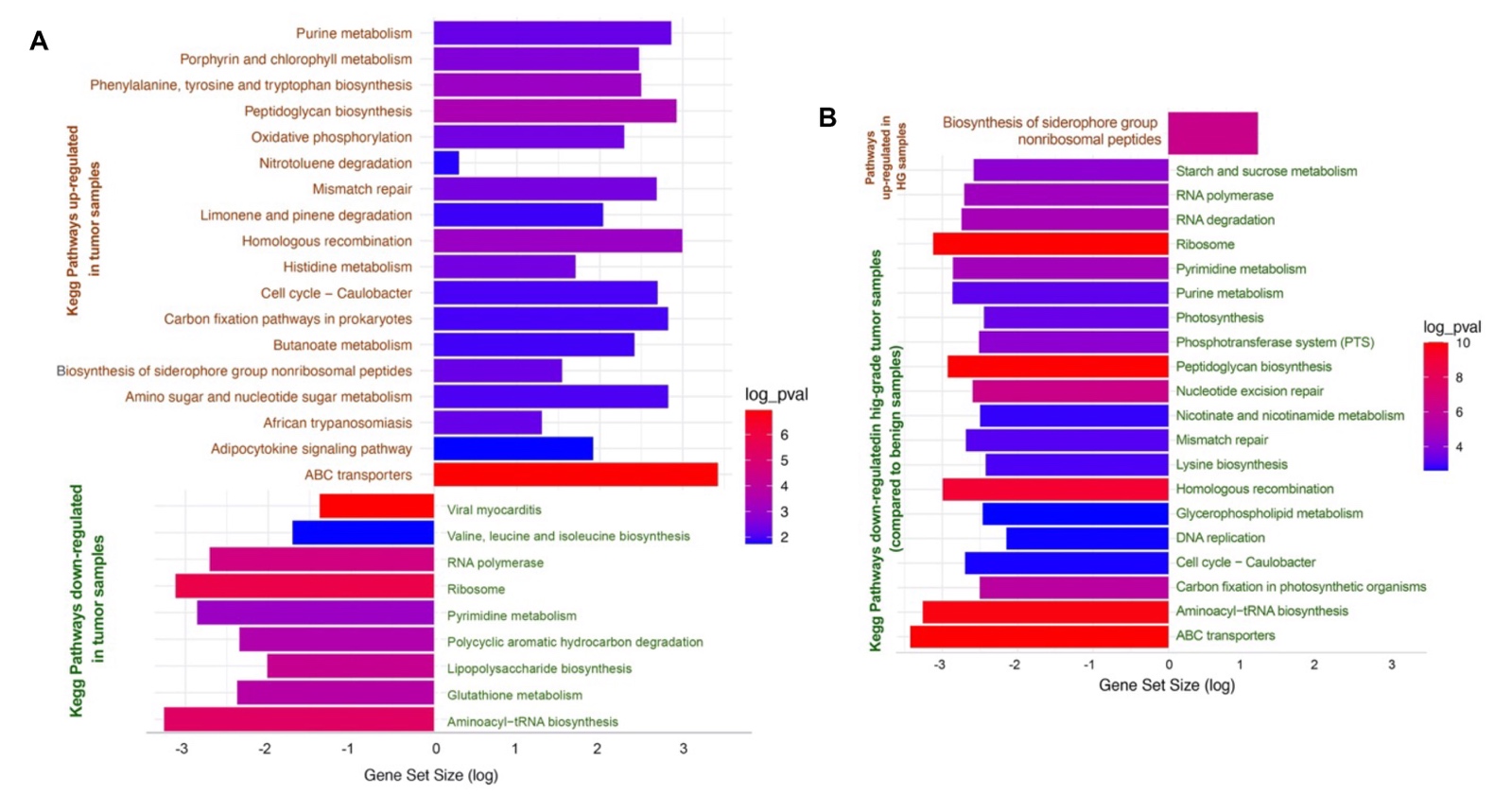


**Supplement 8.** Functional analysis represented by KEGG pathways. **A.** Pathways represented by gene abundance in the metagenomes of endometrial tumors (high-grade (HG) + low-grade (LG)) compared to benign (up or down). **B.** Pathways represented by gene abundance in the metagenomes of HG-endometrial tumors alone compared to benign (up or down). All shown pathways are statistically significant (p<0.05).
