# Supplementary material for: The Vaginal Microbiome is Associated with Endometrial Cancer Grade and Histology
Source: Cancer Res Commun. 2022 Jun 16;2(6):447–55. doi: 10.1158/2767-9764.CRC-22-0075 (PMC9345414; doi:10.1158/2767-9764.CRC-22-0075)
Supplement: Supplement 9 — Biomarker discovery by histology [file crc-22-0075-s09.docx]

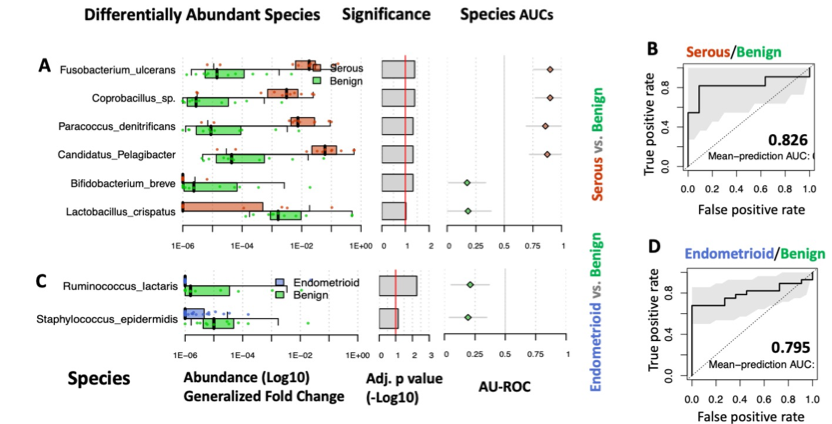


**Supplement 9. Biomarker Discovery by histology.** Additional validation was performed on random forest classifier models, which identified an optimal microbiome signature for each cohort (A, C). These signatures were used to construct receiver operating curves which discriminate serous histology and benign (B), and endometrioid histology versus benign (D).
